# Maternal mental health predicts risk of developmental problems at 3 years of age: follow up of a community based trial

**DOI:** 10.1186/1471-2393-8-16

**Published:** 2008-05-06

**Authors:** Suzanne C Tough, Jodi E Siever, Shirley Leew, David W Johnston, Karen Benzies, Dawne Clark

**Affiliations:** 1 Department of Paediatrics, University of Calgary, Calgary, Alberta, T2T 5C7, Canada; 2 Department of Community Health Sciences, University of Calgary, Calgary, Alberta, T2N 4N1, Canada; 3 Decision Support Research Team, Calgary Health Region, Calgary, Alberta, T2T 5C7, Canada; 4 Faculty of Nursing, University of Calgary, Calgary, Alberta, T2N 1N4, Canada; 5 Child and Youth Studies, Mount Royal College, Calgary, Alberta, T3E 6K6, Canada

## Abstract

**Background:**

Undetected and untreated developmental problems can have a significant economic and social impact on society. Intervention to ameliorate potential developmental problems requires early identification of children at risk of future learning and behaviour difficulties. The objective of this study was to estimate the prevalence of risk for developmental problems among preschool children born to medically low risk women and identify factors that influence outcomes.

**Methods:**

Mothers who had participated in a prenatal trial were followed up three years post partum to answer a telephone questionnaire. Questions were related to child health and development, child care, medical care, mother's lifestyle, well-being, and parenting style. The main outcome measure was risk for developmental problems using the Parents' Evaluation of Developmental Status (PEDS).

**Results:**

Of 791 children, 11% were screened by the PEDS to be at high risk for developmental problems at age three. Of these, 43% had previously been referred for assessment. Children most likely to have been referred were those born preterm. Risk factors for delay included: male gender, history of ear infections, a low income environment, and a mother with poor emotional health and a history of abuse. A child with these risk factors was predicted to have a 53% chance of screening at high risk for developmental problems. This predicted probability was reduced to 19% if the child had a mother with good emotional health and no history of abuse.

**Conclusion:**

Over 10% of children were identified as high risk for developmental problems by the screening, and more than half of those had not received a specialist referral. Risk factors for problems included prenatal and perinatal maternal and child factors. Assessment of maternal health and effective screening of child development may increase detection of children at high risk who would benefit from early intervention.

**Trial registration:**

Current Controlled Trials ISRCTN64070727

## Background

The prevalence of developmental disabilities in North America and Australia is estimated between 12% and 17% [1-3]. Detection and amelioration of developmental problems in the preschool period increases the likelihood that children enter school ready to learn and succeed [4,5]. When developmental problems go undetected and untreated, there is an increased probability of school failure, behaviour problems, low self esteem and loss of potential [6,7]. Thus, undetected and untreated developmental problems can have a significant economic and social impact on society.

Intervention to ameliorate potential developmental problems requires early identification of children at risk of future learning and behaviour difficulties. Because physicians and public health nurses are the primary trusted health professionals in routine contact with children under the age of five, they are ideally positioned to screen children and to identify risk of developmental and behavioral problems [8,9]. Brief clinical observation is the most commonly used strategy for identifying developmental and/or behavioral problems, however, 50 to 70% of children are missed by this method, particularly those with less severe delay [1,10-12]. This level of under-detection by primary care providers may result from a lack of recognition of potential risks to development and lack of effective standard screening as well as limited time, resources and remuneration for screening for developmental problems, follow up and referrals [9,13,14].

The implementation of parent-completed screening tools may assist primary care providers in identifying children who would benefit most from early intervention without adding undue burden on providers [13,15,16]. In general, parent report has equaled or corresponded to test scores used for identification of developmental problems [17], and parent completed tools have demonstrated validity [14].

Evidence also indicates that child development is influenced by family demographics and lifestyle, such as maternal mental health and income [8,18,19]. These are factors that can be identified early on, even prior to birth. Early detection of family, social, and environmental contexts that put children at risk for developmental problems may provide an opportunity to intervene and work with families and communities to create environments that support the optimal development of their children.

Understanding what characterizes at-risk families and children would inform the strategic implementation of community services to support optimal child development. The objectives of this study were to estimate the proportion of children who would screen at risk of developmental problems among preschool children born to medically low risk women, identified by a standardized screening instrument, and to identify parental and environmental factors which were most strongly associated with developmental screening results.

## Methods

### Participants

**Figure 1:**
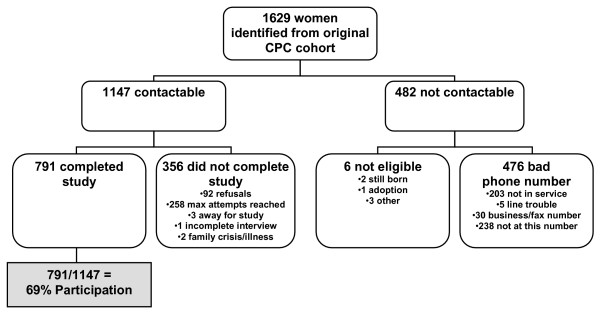
Study flowchart mapping eligibility, recruitment, and completion of mothers who participated in the follow up study.

Medically low risk women, who participated in a randomized controlled trial of supplementary prenatal care between April 2001 and July 2004 and agreed to future research, were invited to participate in this follow-up study. Low medical risk referred to women who did not require prenatal care from an obstetrician, and had an uncomplicated pregnancy such that specialist care for fetal or maternal complications was not required. Pregnant women who sought services provided by family physicians at one of three participating Calgary maternity clinics were included in the study. Women were excluded from the study if they were under the age of 18 (due to ethical issues associated with confidentiality and informed consent), had not completed the baseline study prior to their first appointment with the clinic, did not plan to attend the clinic at the time of the first recruitment call, lived outside the Calgary Health Region, were not pregnant, or could not communicate to study interviewers or translators in one of seven languages (English, French, Cantonese, Mandarin, Punjabi, Urdu, or Arabic dialects) (Figure 1).

In the original trial, women who participated were randomized to: (1) current standard of prenatal care; (2) standard of care plus support from a nurse; or (3) standard of care plus support from a nurse and home visitor. Study participants completed 3 computer-assisted telephone interviews over the perinatal period (first trimester, 32–34 weeks gestation, and 8 weeks post delivery). Data on demographics, lifestyle, psychosocial health, resource utilization, network orientation, and history of abuse and neglect were collected. Of the 2,556 women who were eligible for the study, 1,737 (68%) agreed to participate and completed the first questionnaire. Of those who agreed to participate, 78 percent (1,352/1,737) completed all three questionnaires. Non-completion rates did not differ by study group, but women who did not complete the study tended to be younger, non-Caucasian and had lower education than those who completed the study. Demographic and lifestyle characteristics did not differ by study group. Overall, 75 percent of women in the study were of Caucasian ethnicity and 73 percent of women had some college or university level education. A full description of the randomized controlled trial and results are reported elsewhere [20].

In this follow-up study, mothers were contacted by telephone (using their most recent contact information) when their child was approximately three years old, and they were invited to complete a telephone questionnaire. Before completing the questionnaire, respondents were informed by the interviewers that their participation was voluntary, that their responses would be linked to the original trial data, and that information would be kept confidential. Women who miscarried, did not speak English, did not reside in the city of Calgary any longer, or who had an incorrect phone number were excluded. The follow-up study was launched on November 30, 2005 and was completed on March 27, 2006. The study received ethical approval from the Conjoint Medical Bioethics Committee of the University of Calgary and Calgary Health Region.

### Questionnaire

The telephone questionnaire took approximately 15–20 minutes to complete and included information on child health and medical care, child development, child care, mother's demographics and lifestyle, mother's emotional and physical well-being, and parenting style. Items were generated by the research team in consultation with community partners over a 6 month period at monthly meetings to address the research question and to ensure potential covariates were considered (eg. access to physician services, child care). If an identified construct could be assessed with standardized tools (eg. child development, parenting, social support, detailed below), the research team reviewed and considered appropriate tools and made decisions by consensus. Criteria for tool selection included the psychometric properties of the scale, reading level and length.

The questionnaire was pilot tested with 20 mothers to assess the length, flow, and comprehension. The questionnaire was revised and shortened based on comments from the pilot test as well as expert consultations. Expert consultants included those with backgrounds in: early child development, speech language development, community service delivery, nursing, social work, epidemiology, survey development, biostatistics, and parenting.

### Outcome Measure

The Parents' Evaluation of Developmental Status (PEDS) was used to screen infants for risk of developmental and/or behavioral problems [21]. The PEDS can be used from birth to 8 years of age and is written at a grade 4 to 5 reading level. Validity and reliability has been determined through assessment of more than 771 children in various settings across the US including physician offices, outpatient clinics, day care centers, and schools. In addition, the PEDS has been standardized on 2823 families from a range of economic and ethnic backgrounds.

The PEDS is a 10 item parent report screening measure designed to facilitate parent-professional communication about development and to increase the probability that developmental and behavioral problems in children, birth to 8 years of age, are detected and addressed. This is accomplished by eliciting parental concerns, determining a child's level of risk for problems, and identifying the appropriate next steps. The PEDS classifies children into one of five categories or 'Paths' based on parents' report of concerns: (A) multiple significant concerns are present that are predictive of disability (high risk); (B) one significant concern is present that is predictive of disability (moderate risk); (C) nonsignificant concerns are present that are not predictive of disability but there is elevated risk for behavioral problems (including mental health problems) because of disruption of family functioning, parent-child conflict and/or disciplinary problems (moderate – low risk); (D) parents have problems communicating (moderate risk); or (E) no concerns are present (extremely low – no risk). The PEDS has a sensitivity and specificity that ranges between 70% and 80%, and these percentages increase with repeated administration [21].

### Independent Variables

Potential covariates of interest were grouped into four categories: child characteristics, home environment, sociodemographic factors, and pregnancy-related variables.

### Child Characteristics

Child health status was based on maternal report (excellent, good, fair, poor, or terrible) and on change in the child's health compared to 1 year ago (about the same, better, or worse). Caregivers reported on health care utilization, routine health examinations, immunizations, having a family doctor, chronic conditions, vision and hearing problems, and referrals.

### Home Environment Characteristics

Maternal physical and emotional health status was based on self report (excellent, good, fair, poor, or terrible) [22]. Questions about abuse, social support, and relationship with a partner were asked. Information about parenting was collected and included parenting morale, which was assessed using the Parenting Morale Index [23], and parenting style, which was assessed using two subscales of the National Longitudinal Survey of Children and Youth (hostile/ineffective and aversion) [24].

### Sociodemographic Factors

Self-reported information on marital status, education, annual household income, ethnicity, and lifestyle factors (smoking, alcohol, drug use) was collected.

### Pregnancy-Related Variables

Several variables measured during the original randomized controlled trial were included in this analysis, including the Kellner Symptom Questionnaire [25], Rosenberg Self Esteem [26], McCubbin Social Support Index [27], Woman Abuse Screening Tool (WAST) [28], Edinburgh Postnatal Depression Scale [29] and Vaux Network Orientation Scale [30].

### Statistical Analysis

Data collected from the follow-up study were linked to data from both the original randomized controlled trial using unique research identifiers (to include pregnancy-related variables in the analysis) and to provincial perinatal records (to obtain the most accurate estimate of gestational age of the infant at birth). This resulted in data for mothers at four time points from their first trimester to 3 years post partum.

Bivariate comparisons between the PEDS path and independent variables in the four categories of interest were conducted using a chi-squared test. A multinomial logistic regression model was constructed to explore the relationship between risk of developmental problems and factors associated with this risk, yielding odds ratios and 95% confidence intervals. Statistical significance was set at p < 0.05 for bivariate analyses and was also the criteria for considering variables for regression modeling along with known confounders. Selected predicted probabilities for screening in each PEDS path were also calculated. All statistical analyses were performed using Stata 9/SE version 9.2.

## Results

### Study Response and Demographics

The results of the original randomized control trail indicated that additional support provided by nurses, or nurses and home visitors, could increase the number of women who use existing community based resources and increase the amount of information women obtain about pregnancy related topics. However, rates of alcohol and tobacco use, post partum depression and birth outcomes did not differ by group. The type of prenatal care a women obtained in the original trial was not a predictor of the PEDS score (p = 0.737) and was not controlled for in subsequent analysis.

Mothers from the original trial who could be contacted and agreed to participate (N = 791) represented 69% of those eligible (N = 1147) (Figure 1). Characteristics of mothers and their children at the time of the follow up study are described in Table 1 and reflect a middle income community in a large urban setting. Mothers who could not be contacted or refused to participate were more likely to be less than 25 years old (14% vs. 9%, p = 0.012), non-Caucasian (25% vs. 16%, p < 0.001), smoke during pregnancy (25% vs. 16%, p = 0.001), have required food bank support (6% vs. 3%, p = 0.035), scored low on scales that assess ability to seek help (36% vs. 29%, p = 0.009) and to have scored low on self esteem during pregnancy (30% vs. 23%, p = 0.010) compared to those mothers who completed the questionnaire.

**Table 1:** Characteristics of mothers and children who participated in the follow up study

**Characteristic**	**N = 791** **n**	**%**
**MOTHERS**		
Married/Common law	746	94.4
Education		
High school or lower	124	15.7
College/university/trade	588	74.3
Post graduate studies	79	10.0
Household income per year		
< $40,000	65	8.8
$40,000–$80,000	267	36.0
> $80,000	410	55.3
Caucasian ethnicity	668	84.5
Any smoking in the past month	98	12.5
Any alcohol in the past month	514	65.0
Any drugs in the past month	17	2.2
Excellent or good rating of physical health in the past 6 months	599	75.8
Excellent or good rating of emotional health in the past 6 months	588	74.3
2 weeks or more of self reported depression since infant born	281	35.5
6 months or more of self reported depression since infant born	96	12.2
Edinburgh Post Partum Depression Score >10 within 4 months of delivery	54	8.17
Edinburgh Post Partum Depression Score >13 within 4 months of delivery	20	3.03
Ever seen or witnessed abuse since child was born	105	13.3
Mother has been abused since child was born	50	6.3
		
**CHILDREN**		
Age ≥ 3 years	468	59.2
Male	383	48.4
Born preterm (< 37 weeks)	49	6.6
Child has regular family doctor	750	94.9
A parent stayed home with the child for ≥24 months	320	40.5
Child has had routine health exam	711	90.1
Child's immunization shots are up to date	742	94.0
Child's current general health*		
Excellent/Good	731	92.4
Fair/Poor/Terrible	60	7.6
Child received non-parental care for >20 hours per week in the past 6 months	481	60.8
Parent reads to child once or more per day	696	88.2

Note: Denominator varies due to missing data

* as rated by the child's mother

### Child Development Screening Results

Based on the PEDS, 11% (n = 86) of children screened at high risk (Path A) and 30% (n = 239) screened at moderate risk (Path B) of developmental problems. Twenty four percent screened (n = 186) at an elevated risk for behavioral problems and/or mental health problems (Path C). There were no children who screened at moderate risk because their parents had problems communicating (Path D). Thirty five percent (n = 280) of children screened were not at risk for either developmental or behavioral problems (Path E). Mothers reported concerns with expressive language for 81% of children at high risk of developmental problems (Path A) and for 58% of children at moderate risk of developmental problems (Path B). Mothers also reported behavioral concerns in over half of the children identified at high and moderate risk of developmental problems (Paths A and B) and in 68% of children identified at elevated risk for behavioral and/or mental health problems (Path C).

Among the 86 children who screened at high risk of developmental problems, 43% (n = 37) had previously been referred for further assessment. High risk children most likely to have received a referral had been born preterm (15% vs. 0%), had previously had their hearing tested (70% vs. 31%), and had vision problems (11% vs. 0%, all p < 0.05, Table 2).

**Table 2:** Characteristics of children who had a referral compared to those who did not, among children screened at high risk of developmental problems (Path A)

**Characteristic**	**Referral** **N = 37** **n (%)**	**No referral** **N = 49** **n (%)**	**p-value**
Age ≥ 3 years	22 (59)	34 (61)	0.339
Born preterm	5 (15)	0 (0)	0.006
Male	28 (76)	32 (65)	0.300
Child has regular family doctor	34 (92)	43 (88)	0.726
Child has had routine health exam	34 (92)	46 (94)	1.000
Child's immunization shots are up to date	35 (95)	44 (90)	0.694
Child has had ear infections prior to age 2	24 (65)	22 (45)	0.066
Child has had hearing tested	26 (70)	15 (31)	<0.001
Child has vision problems	4 (11)	0 (0)	0.031
Child's current general health*			
Excellent/Good	28 (76)	45 (92)	0.065
Fair/Poor/Terrible	9 (24)	4 (8)	
Compared to 1 year ago, child's health is:			
About the same	17 (46)	32 (65)	0.191
Better	18 (49)	16 (33)	
Worse	2 (5)	1 (2)	
Child has/had congenital abnormality	2 (5)	1 (2)	0.575
Child has/had chronic breathing problems	6 (16)	4 (8)	0.249
Child has/had allergies	6 (16)	3 (6)	0.165
Child has/had eczema or psoriasis	7 (19)	14 (29)	0.302
Child has/had sleep problems	1 (3)	0 (0)	0.430
Low parenting morale	7 (19)	8 (16)	0.754
Hostile/Ineffective parenting (cut at 10^th ^percentile)	4 (11)	13 (27)	0.101

Note: Denominator varies due to missing data

The chi squared analysis suggested that children at high risk of developmental problems (Path A) were significantly more likely to be male, to have had ear infections (and hearing tests) and to have parents who reported improved child health compared to a year ago (all p < 0.05, Table 3). The most common referral was to a speech and language pathologist and rates of referral increased as children screened at greater risk (p < 0.001). Children who screened at high risk for developmental problems (Path A) were more likely to come from a single parent family and/or lower income homes (p < 0.014). Their mothers were also more likely to report a history of depression (36%), abuse prior to pregnancy (47%), distress during pregnancy (45%), and more than 2 weeks of depression in the post partum period (47%, all p < 0.05). In addition, they were more likely to report tension in their marital relationship (56%, p < 0.004) and less likely to report that their families ate meals together on a daily basis (74%, p < 0.014, Table 3). Mothers of children at high risk for developmental problems were also less likely to report high parenting morale (p < 0.05).

**Table 3:** Characteristics of children and their environment for children in each risk category

	**Path A** (high risk of developmental problems)	**Path B** (moderate risk of developmental problems)	**Path C** (elevated risk of behavioral and/or mental health problems)	**Path E** (extremely low to no risk)	
	**n (%)** **N = 86**	**n (%)** **N = 239**	**n (%)** **N = 186**	**n (%)** **N = 280**	**p-value**

**Child's health history and current health**					
Male infant	60 (70)	124 (52)	88 (47)	111 (40)	<0.001
Ear infections prior to age 2	46 (53)	90 (39)	68 (37)	96 (35)	0.018
Child has problems with vision	4 (5)	8 (3)	6 (3)	8 (3)	0.846
Child's current general health					
Excellent/Very good	73 (85)	215 (90)	177 (95)	266 (95)	0.003
Fair/Poor/Terrible	13 (15)	24 (10)	9 (5)	14 (5)	
Compared to 1 year ago, child's health is*:					
About the same	49 (57)	170 (71)	141 (76)	219 (78)	0.007
Better	34 (40)	62 (26)	38 (20)	55 (20)	
Worse	3 (3)	7 (3)	7 (4)	6 (2)	
Preterm delivery	5 (6)	15 (7)	16 (9)	13 (5)	0.388
					
**Child's health care**					
Child has been referred to:					
Early intervention program	8 (9)	7 (3)	2 (1)	1 (1)	<0.001
Speech and language pathologist	21 (35)	30 (13)	3 (2)	6 (2)	<0.001
Child developmental pediatrician	8 (9)	7 (3)	3 (2)	4 (1)	0.005
Psychologist	3 (3)	2 (1)	0 (0)	0 (0)	0.003
Physiotherapist	7 (8)	8 (3)	3 (2)	9 (3)	0.071
Child has had any referral	37 (43)	58 (24)	22 (12)	35 (13)	<0.001
Child has had hearing tested	41 (48)	67 (28)	58 (31)	53 (19)	<0.001
Child has had hearing tested due to repeat or chronic ear infection	11 (27)	6 (9)	5 (9)	6 (11)	0.028
Hearing tested due to suspected deafness	15 (39)	14 (21)	6 (10)	8 (15)	0.006
					
**Mother's demographics and lifestyle**					
Marital Status					
Married/Common-law	79 (92)	229 (96)	173 (94)	265 (95)	0.014
Separated/Divorced	2 (2)	6 (3)	2 (1)	12 (4)	
Single	5 (6)	4 (2)	10 (5)	3 (1)	
Age < 25 years	2 (2)	11 (5)	13 (7)	9 (3)	0.217
Working at paid job	52 (61)	142 (59)	104 (57)	184 (66)	0.220
Education is high school or lower	18 (21)	40 (17)	28 (15)	38 (14)	0.393
Current household income per year					
<$40,000	13 (16)	19 (8)	15 (8)	18 (7)	0.014
$40,000–$80,000	35 (43)	85 (37)	68 (40)	79 (30)	
>$80,000	34 (41)	123 (54)	89 (52)	164 (63)	
					
**Mother's mental and emotional health history**					
History of depression prior to pregnancy	31 (36)	49 (20)	36 (19)	56 (20)	0.009
Witnessed abuse prior to pregnancy*	46 (53)	86 (36)	76 (41)	98 (35)	0.014
History of abuse prior to pregnancy*	40 (47)	80 (33)	68 (37)	73 (26)	0.003
Poor network orientation during pregnancy	6 (7)	4 (2)	10 (5)	6 (2)	0.024
Feelings of distress during pregnancy	39 (45)	77 (32)	66 (35)	82 (29)	0.043
Feelings of contentment, relaxation, and well-being during pregnancy	43 (50)	155 (65)	139 (75)	207 (74)	<0.001
					
**Mother's mental and emotional health post partum**					
Edinburgh Post Partum Score >10 within first 4 months	9(12)	17 (9)	12 (8)	16 (7)	0.523
Edinburgh Post Partum Score >13 within first 4 months	3(4)	8 (4)	4 (3)	5 (2)	0.659
≥2 weeks of depression since infant born	40 (47)	84 (35)	76 (41)	81 (29)	0.007
Rating of current physical health is fair/poor/terrible	30 (35)	61 (26)	41 (22)	59 (21)	0.058
Rating of current emotional health is fair/poor/terrible	29 (34)	62 (26)	49 (26)	63 (23)	0.217
Currently some tension in relationship with partner	45 (56)	92 (40)	64 (36)	92 (34)	0.004
Rating of current social support is fair/poor/terrible	13 (15)	26 (11)	22 (12)	29 (10)	0.670
					
**Parenting**					
High Parenting Morale	71 (83)	215 (90)	167 (90)	262 (94)	0.024
Parent reads to child once or more per day	74 (86)	211 (88)	164 (89)	247(89)	0.930
Family eats 1 or more meals together daily	64 (74)	207 (87)	147 (79)	240 (86)	0.014

*Types of abuse could include any one of physical, emotional, sexual, financial abuse, or neglect

Note: Denominator varies due to missing data

Among children who screened at risk of behavioral and/or mental health problems (Path C), 41% had mothers who reported post partum depression, and 37% had mothers who reported abuse prior to pregnancy (Table 3). Children who screened at risk of behavioral problems were significantly more likely to have a mother who was single and who had changed partners since the child was born, however, numbers were small (p < 0.03).

Among 219 children who had their hearing tested, 12.8% were tested due to repeat ear infections and 19.6% were tested due to suspected deafness. Children who were referred to a speech language pathologist were significantly more likely to have had their hearing tested (p < 0.001). Among the 60 children who had been recommended for speech and language therapy, 43 (73%) had their hearing tested of which 9 were assessed due to chronic ear infections. About 50% of those referred to speech and language assessment had had at least one ear infection before two years of age.

Multinomial logistic regression analysis revealed that the most significant predictors of screening at high risk (Path A) compared to low risk of developmental problems included male gender, having a history of ear infections prior to age two, a mother with a history of abuse, or a mother with low scores on contentment and relaxation during pregnancy with odds ratios ranging from 1.9 to 3.3 (Table 4). Male gender (Odds Ratio 1.6, 95%, Confidence Interval 1.1–2.3) and low scores on contentment and relaxation during pregnancy (OR 1.5, 95%, CI 1.0–2.2) increased the odds of screening at moderate risk of developmental problems (Path B) (Table 4). Children who screened at risk of behavioral and/or mental health problems (Path C) were more likely to have mothers who had experienced two or more weeks of post partum depression (OR 1.6, 95% CI 1.1–2.4) and who had experienced abuse (OR 1.0, 95% CI 1.0–2.3) (Table 4).

**Table 4:** Multinomial logistic regression of infant and maternal characteristics for developmental delay screening status using the PEDS screening tool.

**Variable**	**Odds Ratio**	**95% C.I.**	**p-value**
**Path A (high risk of developmental problems)**			
Male infant	3.3	(1.9, 5.8)	<0.001
Ear infections prior to age 2	1.9	(1.1, 3.2)	0.019
Household income <$40,000	2.1	(0.9, 4.8)	0.071
Maternal history of abuse	2.2	(1.3, 3.7)	0.006
Low scores on contentment/relaxation during pregnancy	2.5	(1.4, 4.2)	0.001
2 weeks of depression post partum	1.7	(0.9, 2.9)	0.062
**Path B (moderate risk of developmental problems)**			
Male infant	1.6	(1.1, 2.3)	0.011
Ear infections prior to age 2	1.2	(0.8, 1.7)	0.434
Household income <$40,000	1.2	(0.6, 2.4)	0.595
Maternal history of abuse	1.3	(0.9, 2.0)	0.178
Low scores on contentment/relaxation during pregnancy	1.5	(1.0, 2.2)	0.055
2 weeks of depression post partum	1.2	(0.8, 1.8)	0.333
**Path C (elevated risk of behavioral and/or mental health problems)**			
Male infant	1.3	(0.9, 2.0)	0.167
Ear infections prior to age 2	1.1	(0.7, 1.7)	0.655
Household income <$40,000	1.2	(0.6, 2.5)	0.635
Maternal history of abuse	1.5	(1.0, 2.3)	0.060
Low scores on contentment/relaxation during pregnancy	0.9	(0.6, 1.5)	0.845
2 weeks of depression post partum	1.6	(1.1, 2.4)	0.025

Note: Path E (extremely low to no risk) is the comparison (baseline) group

Based on the logistic regression model, a male infant with a history of ear infections, who had a mother with a history of abuse, low scores on relaxation during pregnancy, and at least two weeks of depression post partum had a predicted probability of 53% for screening at high risk of developmental problems (Path A) (Table 5). A similar child, whose only difference was having a mother with a positive history of well-being, had a predicted probability of 19% for screening at high risk of developmental problems. The predicted probability of risk for developmental problems based on child and maternal characteristics is further illustrated in Table 5.

**Table 5:** Predicted probability for each PEDS path from the multinomial logistic regression model.

**Infant characteristics**		**Environment**		**Predicted Probability of Screening in Each PEDS Path**			
**Gender**	**Ear infections**	**Household income**	**Mother with a history of abuse, postpartum depression, and poor contentment during pregnancy**	**Path A** (high risk of developmental problems)	**Path B** (moderate risk of developmental problems)	**Path C** (elevated risk of behavioral and/or mental health problems)	**Path E** (extremely low to no risk)

Male	Ear infections	Low income	History	0.53	0.23	0.15	0.09
Male	Ear infections	High income	None	0.11	0.32	0.22	0.35
Male	Ear infections	Low income	None	0.19	0.32	0.21	0.28
Male	None	Low income	None	0.12	0.32	0.22	0.33
Female	None	High income	None	0.03	0.25	0.22	0.50
Female	None	High income	History	0.12	0.33	0.27	0.28
Female	Ear infections	High income	None	0.04	0.27	0.22	0.47
Female	Ear infections	Low	None	0.08	0.28	0.23	0.41

## Discussion

Over 90% of the children in this study had routine health exams, a family physician and current immunization status. Of these children 10% were identified by PEDS as being at high risk for developmental problems, however over half of them had not received a more detailed assessment by 3 years of age. High risk children most likely to have received a referral were identified by preterm delivery. This study highlights the need for implementation of effective developmental screening to ensure that all children at risk of developmental problems, not just those born preterm, have a similar probability of being identified and of receiving appropriate and timely early intervention.

The variables that were associated with an increased probability of referral in this study (preterm birth, had hearing tested, had vision problems) are medical in nature, but this study, of primarily middle and upper income families under a system of universal health care, shows that sociocultural variables have a significant relationship to the development of children. Those children most at risk of developmental problems were characterized by having mothers with a prenatal history of abuse, depression, distress and an unwillingness to access social support networks as well as fewer financial resources. In the post-partum period, children at risk could be identified by mothers with post partum depression and marital tension. Consequently, identification of children at risk of developmental problems could begin earlier by identifying mothers with poor emotional and social health in the prenatal and early post partum period.

The link between maternal depression, prenatal stress and child development is well established [31-34]. Postpartum depression has been associated with negative maternal attitudes and may adversely influence the mother-infant relationship, increasing the risk for delayed cognitive development and child behavior problems [35-37]. It can also interfere with parenting self-efficacy, parenting skills and marital satisfaction for both partners [38-40]. From this study, a male infant with ear infections, a low income environment, and a mother with a history of psychosocial risk (e.g. history of abuse, postpartum depression, and poor contentment during pregnancy) had a 53% chance of screening at high risk for developmental problems. If there was an absence of psychosocial risk during pregnancy and post partum, this same child would have had a reduction in the likelihood of screening at the same level of risk by over 30%.

Although this study provided a unique opportunity to examine prenatal, post natal and current variables associated with child health and development at age 3, the data are limited in that women who could not be reached at follow up were younger, had lower self esteem and fewer financial resources. Consequently our findings may be best generalized to women over age 25 and families with middle or higher incomes. Furthermore, although the PEDS has a reported sensitivity and specificity of about 80%, which is considered good given the nature and complexity of child development [41], telephone administration of the PEDS has not been well researched. Preliminary findings suggest that the telephone PEDS is reliable but may have reduced sensitivity to identify parents concerns resulting in underestimation of risk [42]. Consequently, the combination of loss in the follow up of our more vulnerable mothers and the use of telephone follow up suggest our estimates of risk of developmental problems may be conservative.

## Conclusion

This study indicates that there are missed opportunities for the screening and identification of children at high risk of developmental problems who may benefit from further assessment. Ethical implications of putting effective standard screening into practice would necessitate follow-up resources and processes, including assessments for approximately twice as many children as are currently being seen, as well as appropriate, readily available, evidence based, early intervention programs [43]. The structural, economic and community resources necessary for ethical screening would require planning and organization, including training and recruitment of speech language technologists and specialists.

Developmental risk for some children may be identified very early by prenatal maternal characteristics such as history of abuse or depression, lack of contentment and well-being, and low annual family income. Prenatal intervention could occur for families that screen at-risk with a focus on maternal mental health and early parenting support. The goal would be the reduction of risk for developmental problems in children in the preschool years. Indeed, the reduction in probability of screening at risk for male infants from 53% to 19% through attention to maternal mental health, with no change in economic circumstance, provides a compelling incentive to address issues of well being, social support and early identification of poor mental health. Attention to antecedent events is in alignment with a Population Health Approach and with the United Nations Convention on the Rights of the Child [44,45]. Furthermore, improving maternal mental health and supports for parenting may have positive influence beyond the index child with regard to improved outcomes for other children in the home and improved marital relations. Based on this, we are implementing a research project that investigates the impact of reorienting prenatal care to address mental health and social support. Ultimately, effective screening of child development in combination with a comprehensive assessment of all aspects of maternal health would increase the detection of children at highest risk of developmental problems who would benefit from early intervention and support for the health of their families.

## Competing interests

The authors declare that they have no competing interests.

## Authors' contributions

SCT conceived of the study and supervised all aspects of its implementation. JES completed the analyses. DWJ managed study implementation and data collection. KB, SL, and DC, contributed to development of the survey instrument and interpretation of findings. All authors helped to conceptualize ideas, interpret findings, and review and revise drafts of the manuscript. All authors read and approved the final manuscript.

## Pre-publication history

The pre-publication history for this paper can be accessed here:


